# Safety, tolerability, pharmacokinetics, and pharmacodynamics of KN060, a humanized anti-FXI/FXIa dual-domain antibody, following single ascending doses in healthy Chinese subjects

**DOI:** 10.1016/j.rpth.2025.103322

**Published:** 2025-12-29

**Authors:** Jie Huang, Jinlong Liu, Yaxin Liu, Yichen Cao, Wei Sun, Yanrong Dong, Yuwei Li, Jing Li, Qian Wu, Xiaoyan Yang, Shuang Yang, Chuanpin Chen, Guoping Yang

**Affiliations:** 1 Department of Pharmaceutics, Xiangya School of Pharmaceutical Sciences, Central South University, Changsha, China; 2 Research Center of Drug Clinical Evaluation of Central South University, Changsha, China; 3 Center of Clinical Pharmacology, the Third Xiangya Hospital, Central South University, Changsha, China; 4 National-Local Joint Engineering Laboratory of Drug Clinical Evaluation Technology, Changsha, China; 5 Suzhou Alphamab Co, Ltd, Suzhou, China

**Keywords:** dual-domain antibody, factor XI, pharmacodynamics, pharmacokinetics, safety

## Abstract

**Background:**

KN060 is a humanized dual-domain antibody targeting coagulation factor XI (FXI) and factor XIa (FXIa) and is currently being developed for the prevention of thromboembolic diseases.

**Objectives:**

This study aims to evaluate the safety, tolerability, pharmacokinetics, and pharmacodynamics of a single intravenous administration of KN060 in healthy Chinese subjects.

**Methods:**

This single-center, randomized, double-blind, placebo-controlled clinical study was conducted in 38 healthy Chinese subjects. The study included 6 dose groups (0.1, 0.3, 1.0, 2.5, 5.0, and 10.0 mg/kg), with KN060 administered via a single ascending-dose regimen.

**Results:**

KN060 demonstrates good tolerability and safety, with no significant treatment-emergent adverse events. A single intravenous infusion of KN060, ranging from 0.1 to 10.0 mg/kg, resulted in an average *T*_max_ ranging from 1.02 to 3.34 hours. For a dose range of 0.3 to 10.0 mg/kg, the average half-life ranged from 122.96 to 146.47 hours. Pharmacokinetic and pharmacodynamic analyses revealed that within the dose range of 0.1 to 10.0 mg/kg, drug concentration was positively correlated with activated partial thromboplastin time and negatively correlated with factor (F)XI activity and free FXI content. Immunogenicity analysis showed that all subjects were negative for antidrug antibodies.

**Conclusion:**

The study results demonstrated that KN060 was well tolerated and safe. Based on pharmacokinetic and pharmacodynamic data, KN060 is a promising humanized dual-domain antibody simultaneously targeting FXI/FXIa for the prevention of thromboembolic diseases.

**Trial Registration:**

https://www.chictr.org.cn, identifier: ChiCTR2200056926; http://www.chinadrugtrials.org.cn, identifier: CTR20222339.

## Essentials

• KN060 is a new humanized dual-domain antibody developed to prevent blood clots by targeting factor (F)XI/FXIa.
• This study tested a single intravenous dose of KN060 in healthy Chinese adults.
• KN060 was safe, well tolerated, and showed predictable drug levels in the body.
• KN060 prolonged blood clotting time, suggesting promise for preventing blood clots.

## 1 Introduction

Thrombosis is a complex and progressive pathological process, clinically classified into venous thromboembolic disease and arterial thromboembolic disease, based on the location of the thrombosis [1]. According to data from the Global Burden of Disease study, ischemic heart disease and stroke together account for approximately 25% of global deaths, making them the leading causes of cardiovascular- and cerebrovascular-related mortality [2]. Stroke, in particular, remains a major cause of both disability and death worldwide, with 12.2 million new cases reported in 2019 [3,4]. Additionally, venous thromboembolism has emerged as the third leading cause of cardiovascular-related mortality, following ischemic heart disease and stroke, highlighting the significant public health burden posed by thrombotic disorders [5].Currently, the prevention and treatment of thrombosis primarily rely on anticoagulation, antiplatelet, and thrombolytic therapies, with anticoagulation being considered the cornerstone of thromboembolic disease management [6]. This approach is also recommended in evidence-based guidelines for the prevention and management of venous thromboembolism [7].

Although traditional anticoagulants and direct oral anticoagulants have demonstrated significant efficacy in clinical practice, they each have limitations and do not fully address all clinical needs. Therefore, developing safer and more convenient anticoagulants with a reduced bleeding risk remains a key focus of current clinical research [[8], [9], [10], [11]].

Factor (F)XI acts as a crucial link between the kinin inhibitory–kinin system and the coagulation pathway during tissue factor–initiated coagulation [12]. Targeting FXI can prevent thrombosis and maintains a normal hemostatic response by inhibiting the amplification of the intrinsic coagulation pathway, while preserving the integrity of the extrinsic (tissue factor) pathway and the common pathway [[13], [14], [15]]. Consequently, targeting FXI may present a lower bleeding risk than existing anticoagulant therapies. Moreover, numerous preclinical and clinical studies have preliminarily demonstrated that targeting FXI not only exerts significant antithrombotic effects but also offers a favorable safety profile, thus opening a promising new avenue for thrombosis treatment [[16], [17], [18]]. Currently, research on FXI targets primarily focuses on antisense oligonucleotides [15], monoclonal antibodies, and small molecule compounds [19]. Notable progress has been made with monoclonal antibodies targeting FXI or FXIa, such as Osocimab (BAY-1213790), Xisomab 3G3 (AB023), Abelacimab (MAA868), and MK2060 (results not yet disclosed). Among these, Osocimab, Xisomab 3G3, and Abelacimab have completed several clinical trials, with results indicating that these drugs are safe and well tolerated [16,[20], [21], [22], [23], [24]].

KN060 is a humanized dual-domain antibody simultaneously targeting FXI/FXIa, developed by Suzhou Alphamab Co, Ltd. To date, KN060 has completed preclinical evaluations of pharmacodynamics, pharmacokinetics, and toxicology. In an arteriovenous shunt thrombosis model using New Zealand rabbits, KN060 exhibited significant antithrombotic effects without markedly affecting prothrombin time or increasing bleeding risk. Single- and multiple-dose intravenous pharmacokinetic studies in Sprague-Dawley rats and cynomolgus monkeys revealed no significant sex-based differences in pharmacokinetic parameters. Additionally, multiple-dose intravenous toxicokinetic studies indicated no apparent drug accumulation within the tested dose range, with only 1 instance of antidrug antibody (ADA) positivity observed in the low-dose group of cynomolgus monkeys. These findings suggest that KN060 has a favorable safety and tolerability profile. The primary objective of this study is to evaluate the safety, tolerability, pharmacokinetics, and pharmacodynamics of KN060 in healthy populations.

## 2 Methods

### 2.1 Subjects

Healthy male and postmenopausal/nonmenstruating female Chinese participants aged 18 to 55 years, with a body mass index between 19 and 26 kg/m^2^ (men weighing ≥50 kg and women ≥45 kg), were included in the study. The primary exclusion criteria included a history of chronic illness or any significant current systemic illness; substance abuse within the past year; hypersensitivity to the study compound; failure to meet the requirements for menopause or menstruation in women; recent participation in other clinical trials; recent over-the-counter use of medications or blood donation; planned surgery with bleeding risk within 4 months after dosing; or a history of bleeding disorders. At the time of study initiation, reproductive toxicity studies of KN060 in animals had not yet been completed. Therefore, in accordance with The International Council for Harmonisation of Technical Requirements for Pharmaceuticals for Human Use guidelines and to mitigate potential risks, women of childbearing potential were excluded from this first-in-human trial until relevant nonclinical reproductive toxicity data become available. Participants were free to withdraw from the study at any time and for any reason. Detailed inclusion and exclusion criteria are provided in Supplementary Material. All participants who received KN060 were males (*n* = 29; age range, 18-65 years; mean age, 26-30 years), while the single female participant (postmenopausal, ≥50 years) received placebo, resulting in a predominance of younger males.

### 2.2 Study design

This study is a randomized, double-blind, placebo-controlled phase I clinical trial. The protocol was approved by the Ethics Committee of the Third Xiangya Hospital of Central South University and registered with the Chinese Clinical Trial Registry (https://www.chictr.org.cn) under registration number ChiCTR2200056926. Additionally, the study was also registered with the Chinese Drug Evaluation website under registration number CTR20222339 (http://www.chinadrugtrials.org.cn). The study was conducted in accordance with the Declaration of Helsinki and Good Clinical Practice requirements. All participants provided written informed consent. A total of 38 healthy Chinese subjects were enrolled, with the grouping and dosing regimen as follows: The trial consisted of 6 dose groups, with KN060 doses adjusted based on body weight at 0.1, 0.3, 1.0, 2.5, 5.0, and 10.0 mg/kg, administered as a single intravenous infusion. The first dose group included 2 subjects, all receiving KN060. The second dose group included 4 subjects, and the third to sixth dose groups each included 8 subjects. The dosing procedure was as follows: in the first dose group, the initial subject was dosed and observed for 72 hours; if no safety issues were observed, the second subject was dosed. For the second to sixth dose groups, the first 2 subjects were randomized 1:1 to receive KN060 or placebo and observed for 72 hours; if no safety issues were observed, the remaining subjects were dosed (in the second dose group, the remaining 2 subjects received KN060; in the third to sixth dose groups, the remaining 6 subjects were randomized 5:1 to KN060 or placebo). Dose escalation to the next group proceeded only after confirming safety and tolerability in the preceding group. The dose range was designed based on KN060’s preclinical studies and clinical data from other anti-FXI monoclonal antibodies. The starting dose of 0.1 mg/kg was determined using the NOAEL from Sprague-Dawley rats (200 mg/kg; maximum recommended starting dose: 0.327 mg/kg) and cynomolgus monkey (100 mg/kg, maximum recommended starting dose: 0.405 mg/kg) toxicology studies, calculated via body surface area conversion with a safety factor of 100. In addition, the lowest dose of KN060 was determined with reference to the first-in-human clinical trial doses of 3 anti-FXI antibodies: Abelacimab, Osocimab, and Xisomab. Therefore, the maximum dose was set with an initial dose of 0.1 mg/kg, using a 2- to 3-fold dose escalation approach and was determined as 10.0 mg/kg with reference to the maximum phase I dose of the investigational drug (Osocimab).

KN060 or placebo was mixed with normal saline to prepare a 100-mL solution, administered via intravenous infusion over 60 ± 10 minutes. The investigational product KN060, provided as an investigational product by Suzhou Alphamab Co, Ltd, and manufactured by Jiangsu Alphamab Biopharmaceuticals Co, Ltd, was stored and handled in accordance with the manufacturer’s instructions.

### 2.3 Pharmacokinetic evaluation

A total of 18 blood collection time points were scheduled in this study: within 30 minutes before the intravenous infusion, 30 minutes after the start of the infusion, 5 minutes after the end of the infusion, and at 2, 4, 6, 8, 12, 24, 48, 96, and 144 hours after the start of the infusion, as well as on days 14, 21, 28, 35, 42, and 56. Among them, from the second dose group, the sampling point of 6 hours was added; From the fifth dose group, blood collection points on day 21 and day 35 were added. At each time point, 3 mL of blood was collected and immediately centrifuged at 2 to 8 °C. The concentration of KN060 was measured using an ELISA assay fully validated according to the latest regulatory requirements by Accurant BioTechnology Co, Ltd. The ELISA method used hFXI-Chis (batch number: PC20211229), 22KNR1-M0035 (batch number: 20221202B002), and Bio-iFE166 (batch number: Bio-iFE166-230822LZ) as assay reagents. The KN060 drug substance (batch number: 201020DS) was provided by Suzhou Alphamab Co, Ltd; hFXI-Chis by Suzhou Alphamab Co, Ltd; 22KNR1-M0035 by Shanghai Biointron Biotechnology Co, Ltd; and Bio-iFE166 by Accurant BioTechnology Co, Ltd. The analytical instrument is a Microplate Reader (Molecular Devices). Both the intra-assay and interassay coefficients of variability were <20%.

### 2.4 Pharmacodynamic evaluation

The pharmacodynamic (PD) biomarker analysis included activated partial thromboplastin time (APTT), FXI activity, and free FXI. Blood samples during days −14 to −1 (screening period) were collected via a single venous puncture, samples during days 1 to 7 (inpatient period) were collected via an indwelling intravenous catheter, and samples during days 8 to 56 (follow-up period) were collected via a single venous puncture, to minimize subject discomfort and ensure efficient blood collection. At each time point, 3 mL of blood was collected into tubes containing 3.2% sodium citrate as the anticoagulant and immediately centrifuged at 2 to 8 °C.The specific blood sampling time points and volumes are detailed in Supplementary Table 4. FXI activity is measured using the FXI activity assay kit produced by Siemens Healthineers. APTT, as a key indicator for evaluating coagulation function, is measured using the optical method (based on turbidity change detection). Free FXI was measured using an ELISA assay fully validated according to the latest regulatory requirements by Accurant BioTechnology Co, Ltd. The ELISA method used iFE163-Ld-Fc (batch number: TE20211203), hFXI-Chis (batch number: PC20211229), and Bio-iFE35-Ld-Fc (batch numbers: BioiFE35200422JLX and BioiFE35120822JLX) as assay reagents. iFE163-Ld-Fc and hFXI-Chis were provided by Suzhou Alphamab Co, Ltd, and Bio-iFE35-Ld-Fc was supplied by Accurant BioTechnology Co, Ltd. The analytical instrument is a Microplate Reader (Molecular Devices). Both the intra-assay and interassay coefficients of variability were <20%.

### 2.5 Safety and tolerability evaluation

The safety and tolerability of the drug were assessed by recording the occurrence, severity, and relationship of adverse events (AEs) during the clinical study period. This assessment was complemented by other safety monitoring indicators, including vital signs, 12-lead electrocardiogram, physical examination, clinical laboratory test results, and concomitant medications. AEs were coded and adjudicated by the principal investigator and clinician according to the Medical Dictionary for Regulatory Activities (version 24.0) and the National Cancer Institute Common Terminology Criteria for AEs (CTCAE; version 5.0). All AEs were continuously monitored until symptoms returned to normal or a stable condition.

### 2.6 Immunogenicity evaluation

Immunogenicity samples were collected at 6 time points: before administration, at 144 hours, as well as on days 14, 28, 42, and 56 postadministration. At each time point, 3 mL of blood was collected and immediately centrifuged at 2 to 8 °C. Plasma anti-KN060 antibody were detected by Accurant BioTechnology Co, Ltd, using an electrochemiluminescence assay. The assay uses a bridging format with biotinylated and ruthenylated KN060 conjugates to detect ADA in plasma samples. The procedure consists of 3 steps: an initial screening assay to identify potentially positive samples, a confirmatory assay to rule out false positives, and a titer analysis for confirmed positive samples to quantify ADA levels. The assay was fully validated in accordance with current regulatory requirements, with a sensitivity of 14.79 ng/mL. The detailed assay principle is provided in the Supplementary Materials under Principle of Electrochemiluminescence Assay for Anti-KN060 Antibody Detection.

### 2.7 Statistical analysis

Pharmacokinetic parameters were estimated using noncompartmental analysis (NCA) in WinNonlin8.2 and the CPhaMAS platform (version 1.0; https://cphamas.com/) [25], including area under the concentration-time curve from time zero to the last measurable concentration (AUC_0-t_), area under the concentration-time curve from time zero extrapolated to infinity (AUC_0-∞_), mean residence time extrapolated to infinity (MRT_0-∞_), time to reach maximum plasma concentration (*T*_max_), maximum observed plasma concentration (*C*_max_), elimination half-life (*T*_1/2_), clearance, apparent volume of distribution (*V*_d_), percentage of AUC extrapolated beyond the last measured point (AUC__% Extrap_), terminal elimination rate constant (λz), and others. Noncompartmental analysis is a model-independent method that calculates parameters such as AUC and *C*_max_ based on observed blood drug concentration–time data, without assuming a specific compartmental model. All pharmacokinetic parameter definitions have been clearly provided in the Supplementary Materials for readers’ reference and better understanding. The average plasma drug concentration–time curve for each dose group was plotted according to the planned blood collection times. Statistical analysis was performed using SAS 9.4 software. A power model was applied to evaluate the linear relationship between key pharmacokinetic parameters (*C*_max_, AUC_0-t_, and AUC_0-∞_) and the administered dose across each dose range. PD evaluation was conducted using SAS 9.4 software. Individual PD data were collected, and descriptive statistics were performed on the levels of APTT, FXI activity, and free FXI according to dose group. The average drug efficacy–time curves for each dose group were plotted according to the planned blood collection times. Pearson correlation or Spearman rank correlation analysis is used to estimate the correlation between PD biomarkers and plasma concentrations.

## 3 Results

### 3.1 Subjects

**Figure 1:**
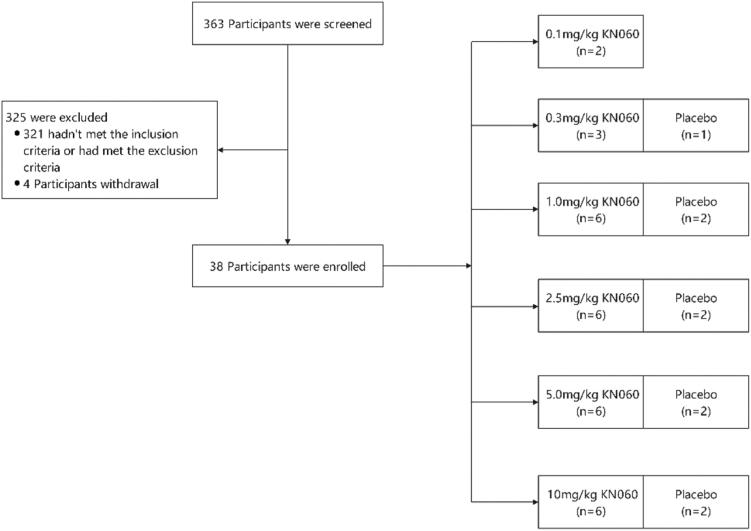
Flow chart of study: 38 volunteers were treated with placebo or KN060 in the 0.1- to 10-mg/kg dose group. The high exclusion rate was primarily due to the study being conducted during the COVID-19 pandemic, with the majority of excluded participants having abnormal test results related to COVID-19.

A total of 363 participants were screened in this study, of whom 321 were not selected. Forty-two participants were randomized, 4 (9.5%) of whom did not receive the trial drug, while 38 (90.5%) received the assigned dose without discontinuation. All subjects were assigned to 1 of 6 dose groups, and the study flowchart is shown in Figure 1. Among the 38 participants, 37 (97.4%) were males and 1 (2.6%) was female. Table 1 summarizes the main demographic characteristics of the study population, with participants receiving KN060 or placebo showing similar demographic profiles.

**Table 1:** Baseline demographic data of the study population.

Characteristic	Dose cohort (mg/kg)						Placebo (*n* = 9)
	0.1 (*n* = 2)	0.3 (*n* = 3)	1.0 (*n* = 6)	2.5 (*n* = 6)	5.0 (*n* = 6)	10.0 (*n* = 6)	
Age (y)							
Mean (SD)	29.5 (4.95)	28.0 (6.08)	29.0 (5.55)	26.3 (5.05)	26.0 (4.60)	26.0 (8.32)	28.4 (11.02)
Minimum to maximum	26-33	24-35	23-37	21-34	20-32	19-40	19-52
Sex(n)							
Male	2/0	3	6	6	6	6	8
Female	0	0	0	0	0	0	1^a^
Weight(kg)							
Mean (SD)	55.65 (2.33)	65.70 (0.95)	62.97 (4.13)	67.63 (10.72)	68.43 (7.01)	63.02 (5.32)	63.44 (5.32)
Minimum to maximum	54-57.3	65.1-66.8	55.7-66.7	57.1-80.5	60.4-79.3	56.3-72.5	57.7-75.1
BMI (kg/m^2^)							
Mean (SD)	20.55 (0.07)	22.43 (0.47)	22.42 (1.32)	23.00 (1.75)	23.55 (1.94)	22.78 (1.63)	22.12 (1.53)
Minimum to maximum	20.5-20.6	21.9-22.8	20.8-24	20.7-24.9	20.8-25.4	20.8-24.9	19.8-25.2

BMI, body mass index.

a Indicates the single female volunteer.

### 3.2 Pharmacokinetics

**Figure 2:**
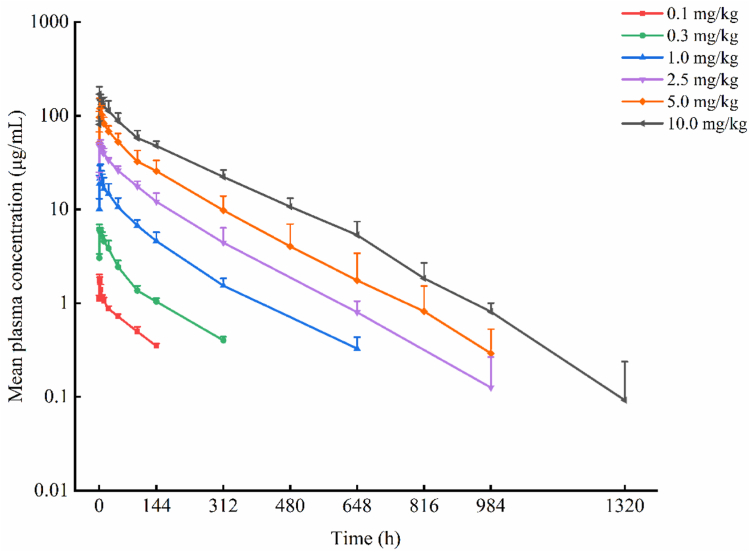
Mean plasma concentration-time curves of KN060 for different dose groups ranging from 0.1 to 10 mg/kg (semilogarithmic scale).

Pharmacokinetic results indicated that the mean *T*_max_ ranged from 1.02 to 3.34 hours at doses of 0.1 to 10.0 mg/kg. Within the dose range of 0.3 to 10.0 mg/kg, the mean half-life varied between 122.96 and 146.47 hours. The mean plasma concentration–time curves (semi-logarithmic plot) for each dose group of KN060 are presented in Figure 2, and the linear plot is provided in Supplementary Figure 1. The primary pharmacokinetic parameters are summarized in Table 2. The linear relationships between pharmacokinetic parameters (*C*_max_, AUC_0-t_, and AUC_0-∞_) and the administered dose were evaluated across the dose range from 0.1 to 10.0 mg/kg. Dose linearity was assessed across the 0.1- to 10.0-mg/kg range. The linearity judgment interval for AUC0-∞ was (0.9364-1.0636), while the dose linearity judgment intervals for *C*_max_ and AUC_0-t_ were (0.9515-1.0484). Within the dose range of 0.1 to 10.0 mg/kg, the 90% CIs of the slope β between the dose of KN060 and the exposure (*C*_max_ and AUC_0-∞_) were (0.94-1.04) and (0.99-1.10), respectively, partially crossing the judgment interval. Further exploratory analysis indicated that within the dose range of 1.0 to 10.0 mg/kg, the linearity judgment interval for *C*_max_, AUC_0-t_, and AUC_0-∞_ was (0.9030-1.0969). The analysis results showed that the relationship between the dose and *C*_max_ of KN060 was basically linear, and the 90% CI of the slope β (0.86-1.05) was almost entirely within the judgment interval. Meanwhile, the 90% CIs of the slope β between the dose of KN060 and the exposure (AUC_0-t_ and AUC_0-∞_) were (0.92-1.08) and (0.91-1.07), respectively, both within the linear judgment interval, indicating that dose and exposure were linearly related within this range. The results of the linearity analysis between pharmacokinetic parameters and KN060 dosage are shown in Supplementary Table 1.

**Table 2:** Main pharmacokinetic parameters of KN060 in healthy Chinese subjects after single intravenous doses.

PK parameters	Dose cohort (mg/kg)					
	0.1 (*n* = 2)	0.3 (*n* = 3)	1.0 (*n* = 6)	2.5 (*n* = 6)	5.0 (*n* = 6)	10.0 (*n* = 6)
*C*_max_ (μg/mL)						
Mean (SD)	1.80 (0.24)	6.20 (0.74)	22.27 (8.56)	50.80 (4.86)	125.14 (33.32)	178.53 (27.11)
RSD (%)	13.2	11.8	38.4	9.6	26.6	15.2
*T*_max_ (h)						
Mean (SD)	1.02 (0.02)	2.34 (1.51)	3.01 (2.00)	2.67 (1.51)	3.34 (2.07)	2.83 (2.79)
RSD (%)	2.3	64.5	66.3	56.5	61.7	98.4
AUC_0-t_ (h × μg/mL)						
Mean (SD)	95.49 (8.41)	457.23 (59.86)	2236.10 (470.47)	5768.43 (1056.83)	11,839.97 (3624.33)	22,401.82 (3635.03)
RSD (%)	8.8	13.1	21.0	18.3	30.6	16.2
AUC_0-∞_ (h × μg/mL)						
Mean (SD)	—	528.39 (64.32)	2299.22 (489.86)	5841.41 (1046.78)	11,915.51 (3660.24)	22,513.74 (3593.98)
RSD (%)	—	12.2	21.3	17.9	30.7	16.0
MRT _0-∞_ (h)						
Mean (SD)	—	140.75 (7.92)	147.84 (11.81)	151.84 (26.30)	156.26 (24.00)	193.60 (12.09)
RSD (%)	—	5.6	8.0	17.3	15.4	6.2
*T*_1/2_ (h)						
Mean (SD)	—	122.96 (5.44)	131.93 (10.48)	127.97 (20.32)	146.47 (8.30)	141.75 (8.23)
RSD (%)	—	4.4	7.9	15.9	5.7	5.8
CL (mL/h)						
Mean (SD)	—	37.60 (4.68)	28.15 (4.83)	29.19 (4.31)	30.17 (7.12)	28.39 (4.06)
RSD (%)	—	12.4	17.1	14.8	23.6	14.3
*V*_d_ (L)						
Mean (SD)	—	6.68 (1.02)	5.36 (1.04)	5.37 (1.13)	6.34 (1.37)	5.80 (0.85)
RSD (%)	—	15.2	19.3	21	21.5	14.5

AUC_0-t_, area under the concentration-time curve from zero to the last measurable concentration; AUC_0-∞_, area under the concentration-time curve from zero to infinity; *C*_max_, maximum drug concentration in plasma; CL, clearance; *T*_max_, time to C_max_; *T*_1/2_, terminal elimination half-life; *V*_d_, apparent volume of distribution; RSD, relative SD.

### 3.3 Pharmacodynamics

**Figure 3:**
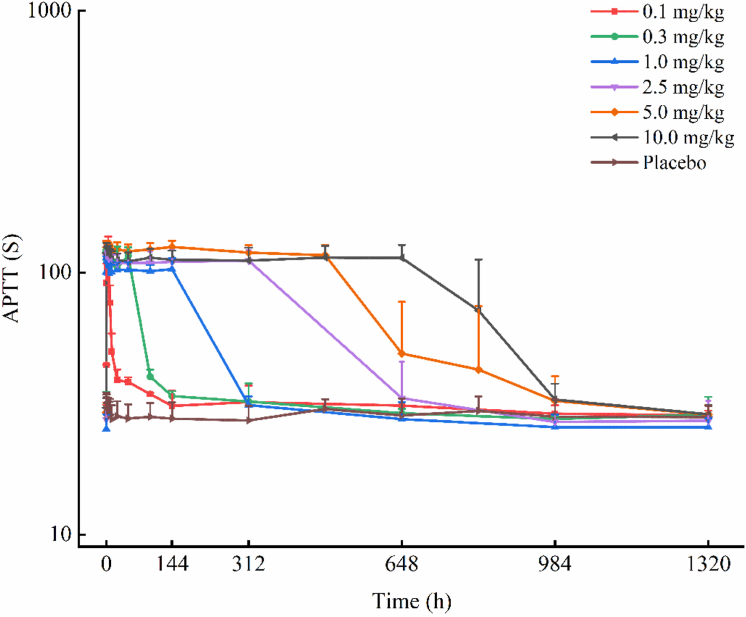
Mean APTT-time curves for KN060 and placebo in different dose groups (semilogarithmic scale). Healthy subjects received a single intravenous infusion of KN060 at doses ranging from 0.1 to 10.0 mg/kg. APTT, activated partial thromboplastin time.

**Figure 4:**
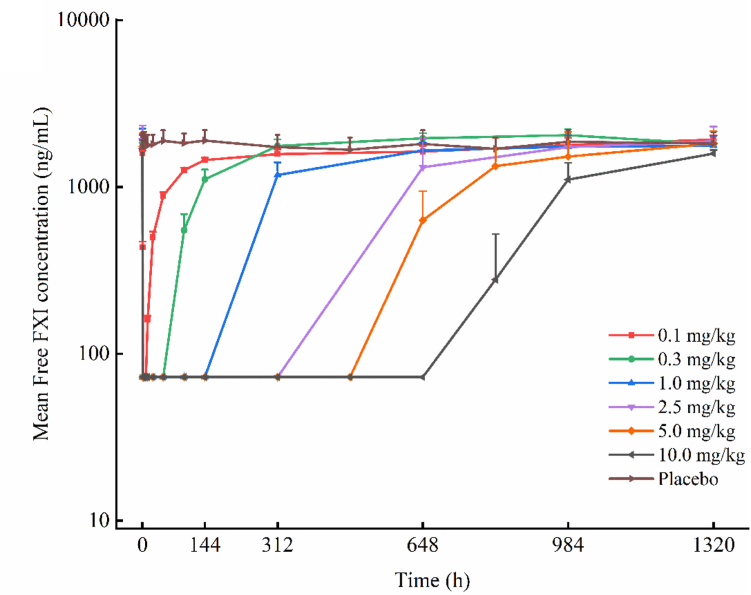
Mean free FXI–time curves for KN060 and placebo in different dose groups (semilogarithmic scale). Healthy subjects received a single intravenous infusion of KN060 at doses ranging from 0.1 to 10.0 mg/kg. In all dose groups (except the placebo group), free FXI levels dropped to below quantification limit within 30 minutes after the start of the infusion. Below quantification limit concentrations were assigned the lower limit of quantification value (72.7 ng/mL).

The mean APTT time curve and mean free FXI time curve (semi-logarithmic plots) for the different dose groups are shown in Figures 3 and 4, respectively. The corresponding linear plots are provided in Supplementary Figures 2 and 3. The mean FXI activity time curve is presented in linear and semilogarithmic scales in Supplementary Figures 4 and 5, respectively. No significant changes were observed in the PD indicators in the placebo group following administration. In each KN060 dose group (1.0-10.0 mg/kg), 30 minutes after the initiation of intravenous infusion, APTT was significantly prolonged to approximately 4 times its baseline value, free FXI was significantly reduced below the detection limit, and FXI activity was inhibited to <5%. With increasing dose, the degree of APTT prolongation did not increase further; however, the effects on APTT, free FXI, and FXI activity were maintained in a dose-dependent manner. In the 10.0-mg/kg dose group, the maximum changes in these three parameters persisted until day 28, after which they gradually returned to baseline levels. Correlation analysis was performed using Pearson correlation or Spearman rank correlation. The data in Supplementary Table 2 reflect that positive values indicate a positive correlation (eg, with APTT), while negative values indicate a negative correlation (eg, with free FXI and FXI activity). We further note that PD indicators reach their maximum change once the drug concentration increases to a certain level; beyond this point, further increases in concentration do not enhance the PD effect but rather extend the duration of the maximum change. Consequently, as doses increase across groups, the correlation strength tends to weaken, which explains the observed variation in correlation coefficients.

### 3.4 Safety and immunogenicity analysis

In this trial, the statistics for AEs related to the trial drug (treatment-related adverse events [TRAEs]) include AEs not only possibly related, probably related, and definitely related to the trial drug but also possibly unrelated to the trial drug, thus increasing the incidence of TRAEs.

In the KN060 group, 26 participants (89.7%) experienced TRAEs, of which 5 (17.2%) were classified as CTCAE grade 2. In the placebo group, 8 participants (88.9%) experienced TRAEs, of which 1 (11.1%) was classified as CTCAE grade 2. There were no TRAEs with CTCAE of ≥grade 3, leading to interruption/adjustment of medication, leading to discontinuation of medication, leading to withdrawal from the trial, or leading to death in each KN060 group and the placebo group. In this trial, there was 1 (2.6%) AE with CTCAE grade 3, which occurred in the KN060 1.0-mg/kg group and was lumbar vertebral fracture. It led to hospitalization and was also the only 1 (2.6%) severe AE in this trial; it was definitely unrelated to KN060. No severe AEs or AE of special interest related to the trial drug occurred in this trial. A summary of AEs by severity and type for each group is provided in Supplementary Table 3.

The TRAEs in the KN060 group primarily included various examination abnormalities (25 cases, 86.2%) and infections (2 cases, 6.9%), manifested by increased blood triglycerides, elevated serum uric acid, increased fibrin D-dimer, proteinuria, elevated C-reactive protein, and positive urine occult blood. These events were not associated with significant clinical symptoms. The types and incidence of TRAEs in the KN060 group were similar to those in the placebo group with various examination abnormalities (8 cases, 88.9%) and infections (1 case, 11.1%). No AEs of particular concern related to the KN060 dose were observed. Details are provided in Table 3. Overall, a single intravenous infusion of KN060 (0.1-10.0 mg/kg) demonstrated a favorable safety and tolerability profile in healthy subjects. All subjects were ADA negative.

**Table 3:** The top 10 treatment-related adverse events and treatment-emergent adverse events after treatment.

System organ class preferred term	0.1 mg/kg (*N* = 2)	0.3 mg/kg (*N* = 3)	1.0 mg/kg (*N* = 6)	2.5 mg/kg (*N* = 6)	5.0 mg/kg (*N* = 6)	10.0 mg/kg (*N* = 6)	Placebo (*N* = 9)	Total (*N* = 38)
Various examinations	1 (50.0)	3 (100.0)	5 (83.3)	6 (100.0)	5 (83.3)	6 (100.0)	8 (88.9)	34 (89.5)
Elevated blood triglycerides	0	1 (33.3)	1 (16.7)	1 (16.7)	2 (33.3)	1 (16.7)	3 (33.3)	9 (23.7)
Elevated serum uric acid	0	1 (33.3)	0	3 (50.0)	0	2 (33.3)	2 (22.2)	8 (21.1)
Proteinuria	1 (50.0)	1 (33.3)	1 (16.7)	0	2 (33.3)	0	2 (22.2)	7 (18.4)
Elevated fibrin D-dimer	0	0	2 (33.3)	2 (33.3)	1 (16.7)	1 (16.7)	1 (11.1)	7 (18.4)
Elevated C-reactive protein	1 (50.0)	1 (33.3)	1 (16.7)	0	0	2 (33.3)	1 (11.1)	6 (15.8)
Positive urine occult blood	0	0	0	2 (33.3)	1 (16.7)	1 (16.7)	1 (11.1)	5 (13.2)
Positive occult blood^a^	0	2 (66.7)	2 (33.3)	0	0	0	1 (11.1)	5 (13.2)
Elevated vitamin C^b^	0	1 (33.3)	1 (16.7)	1 (16.7)	0	0	2 (22.2)	5 (13.2)
Positive urinary leukocytes	0	0	0	1 (16.7)	1 (16.7)	1 (16.7)	0	3 (7.9)
Positive bacterial test^b^	1 (50.0)	0	0	0	1 (16.7)	0	1 (11.1)	3 (7.9)
Elevated blood lactate dehydrogenase	0	0	1 (16.7)	2 (33.3)	0	0	0	3 (7.9)
Elevated blood creatine phosphokinase	0	0	0	1 (16.7)	0	1 (16.7)	1 (11.1)	3 (7.9)
Decreased blood glucose	1 (50.0)	0	0	0	0	2 (33.3)	0	3 (7.9)
Infectious and infestational diseases	0	0	1 (16.7)	0	0	1 (16.7)	1 (11.1)	3 (7.9)
Upper respiratory tract infection	0	0	1 (16.7)	0	0	1 (16.7)	1 (11.1)	3 (7.9)

Values are *n* (%).Incidence is calculated using *N* as the denominator.

*N*, number of subjects in each group; *n*, number of subjects in each group meeting specific conditions.

a From fecal samples.

b From urine samples.

## 4 Discussion

This first-in-human clinical study evaluated the safety, tolerability, pharmacokinetics, and pharmacodynamics of KN060 in healthy Chinese subjects following a single intravenous dose. KN060 is a humanized dual-domain antibody simultaneously targeting FXI/FXIa composed of 2 anti-FXI/FXIa single-domain antibodies (huFE56huv1 and huFE97huv1), which are linked in tandem and conjugated to a wild-type human IgG1-Fc fragment. Moreover, huFE56huv1 and huFE97huv1 specifically bind to the Apple2 and Apple3 domains of human FXI, respectively. *In vitro* studies have demonstrated that KN060 does not significantly affect FXIa activation of FIX in the intrinsic coagulation pathway, suggesting that KN060 may carry a lower bleeding risk [[13], [14], [15]].

Currently, 3 anti-FXI monoclonal antibodies—Abelacimab, Osocimab, and Xisomab—have been publicly disclosed, all of which inhibit the intrinsic coagulation pathway through distinct mechanisms of action. Osocimab induces structural rearrangement by interacting with FXIa, while Xisomab prevents FXI activation by activated FXII (FXIIa), thereby inhibiting the reciprocal activation of FXIa and FXII, without affecting FIX activation by FXIa or FXI activation by thrombin. In contrast, Abelacimab exerts its effects by binding to both FXI and its activated form, FXIa [20,23,24]. Similarly, KN060 binds to both FXI and FXIa to exert its effects, but with differing affinities. The equilibrium dissociation constants (*K*_D_) of Abelacimab for human FXI and FXIa are 1.3 × 10^-12^ M and 4.7 × 10^-12^ M, respectively, while those of KN060 are 1.722 × 10^-13^ M for FXI and 1.88×10^-9^ M for FXIa [20]. The shorter half-life of KN060 (5-6 days), compared with Abelacimab (25-30 days), can be explained by its molecular structure as a single-domain antibody–Fc fusion protein, which typically exhibits reduced half-life relative to monoclonal antibodies, and by its higher affinity for FXI. The latter may promote the formation of large immune complexes that are cleared more rapidly from the circulation, thereby contributing to the shorter half-life of KN060.

The results of the trial demonstrated that a single intravenous infusion of KN060 (0.1-10.0 mg/kg) exhibited good safety and tolerability in healthy participants. As consistent with the currently disclosed information for similar drugs, no hypersensitivity reactions, signs of bleeding, or infusion reactions were observed with KN060. Among the 29 participants treated with KN060, 1 tested positive for ADAs at baseline and after partial dosing. However, the ADA titers in the postdose samples were generally consistent with baseline levels, suggesting that the positive ADA results postdosing were not caused by the drug.

In the dose range of 1.0 to 10.0 mg/kg, *C*_max_, AUC_0-t_ and AUC_0-∞_ were linearly related to the administered dose, suggesting that the metabolism or excretion of KN060 is unlikely to be saturated at higher doses, and *in vivo* exposure to the drug is predictable. The linear relationship was not observed in the lower dose range (0.1 and 0.3 mg/kg), which may be attributed to the small sample sizes in the low-dose groups (*n* = 2 for the 0.1-mg/kg group and *n* = 3 for the 0.3-mg/kg group) or to significant individual variability in the clinical data.

After intravenous administration of KN060, APTT was significantly prolonged, reaching approximately 4 times the baseline value. *In vitro* studies revealed that KN060, Xisomab, and Osocimab, at a concentration of 10 μg/mL, extended APTT by 3.71-fold, 2.09-fold, and 2.22-fold, respectively, compared with untreated standard human plasma. Phase 1 studies indicated that Xisomab and Abelacimab prolonged APTT by approximately 2-fold. These findings suggest that KN060 exerts a more potent anticoagulant effect than the other 3 agents. Patients with congenital FXI deficiency typically exhibit mild bleeding tendencies, with spontaneous hemorrhage being rare, and have a lower incidence of ischemic stroke and deep vein thrombosis. A study on FXI-deficient individuals in Israel reported a significant reduction in ischemic stroke incidence among patients with severe FXI deficiency [17]. This finding provides theoretical support for the development of monoclonal antibodies targeting FXI or FXIa, highlighting their primary advantage. The results of this study preliminary evidence supporting KN060’s potential to prolong APTT with a manageable bleeding risk profile. Although no significant bleeding risk was observed in this study, the sample size was relatively small, and further validation of safety is required in larger-scale clinical trials. In the 10.0-mg/kg dose group, the maximum APTT prolongation effect persisted until day 28, highlighting KN060’s potential as a long-acting anticoagulant.

This study has several limitations. First, the small sample sizes typically used in phase I trials limit the ability to adequately detect rare AEs. Therefore, larger clinical trials are needed to more accurately assess the safety of KN060. Second, healthy subjects do not fully represent the actual patient population, and potential pharmacokinetic differences should be carefully considered when using KN060. This is particularly relevant for older patients and those with comorbidities, as their PK profiles may differ from the young, healthy cohort studied here, and related bleeding events should be closely monitored in clinical practice. Additionally, sex imbalance is another limitation of this study, with only 1 female participant included, who was randomly assigned to the placebo group. This imbalance primarily stemmed from the restrictive postmenopausal/nonmenstruating inclusion criterion, as women of childbearing potential were excluded because reproductive toxicity studies in animals had not yet been completed at the time of study initiation. Although preclinical data indicate no significant sex-based differences in the pharmacokinetic parameters of KN060, this sex imbalance may still limit the generalizability of the study results to female populations. It is therefore recommended that future phase II clinical trials include more female participants to further evaluate the safety and PD properties of KN060 across different sexes. Moreover, subsequent clinical trials should only proceed once reproductive toxicity data become available, ensuring adequate protection for women of childbearing potential.

## 5 Conclusion

In summary, this study evaluated the safety, tolerability, pharmacokinetics, and pharmacodynamics of KN060 injection in healthy Chinese subjects. The results of this clinical study provide a foundation for further research on KN060 in the patient population.

## Supplementary material

Supplementary Material

CONSORT Diagram
