# Supplementary material for: Safety, tolerability, pharmacokinetics, and pharmacodynamics of KN060, a humanized anti-FXI/FXIa dual-domain antibody, following single ascending doses in healthy Chinese subjects
Source: Res Pract Thromb Haemost. 2025 Dec 29;10(1):103322. doi: 10.1016/j.rpth.2025.103322 (PMC12887396; doi:10.1016/j.rpth.2025.103322)
Supplement: Supplementary Material [file mmc1.docx]

**Inclusion criteria:**

1. Healthy male or postmenopausal/non-menstruating female subjects;
2. Aged between 18 and 55 years (inclusive) at the time of signing informed consent;
3. Body mass index (BMI) between 19.0 and 26.0 kg/m² (exclusive of cut-off values); male weight ≥ 50.0 kg, female weight ≥ 45.0 kg;
4. Normal APTT, PT, INR, and platelet values;
5. Ability to understand the study protocol and willingness to accept and comply with its requirements.

**Exclusion criteria：**

1. Individuals with a history of chronic or systemic diseases, including but not limited to cardiovascular, respiratory, endocrine and metabolic, urinary, digestive, blood, autoimmune, nervous system, or psychiatric disorders, as well as bacterial or viral infections;
2. Allergic constitution, or allergic to the trial drug/similar drugs or excipients, or with a history of multiple allergic diseases (two or more), or those with a history of specific reactions to certain substances;
3. Female subjects with natural (spontaneous) amenorrhea less than 12 months prior to screening，or female subjects with natural (spontaneous) amenorrhea for 12 months or longer but had serum follicle stimulating hormone (FSH)≤40 U/L;
4. Individuals with a history of drug abuse or dependence, or a history of drug use within the past year;
5. Individuals with a history of alcoholism or regular alcohol consumption in the six months preceding the trial(defined as more than 14 units of alcohol per week, where 1 unit = 360 ml of beer, 45 ml of 40% alcohol spirits, or 150 ml of wine); or those who cannot abstain from alcohol during the trial;
6. Smoke more than 10 cigarettes a day within 3 months, or who cannot comply with the regulations of the study center while staying in the Phase I clinical study ward;
7. Have donated blood or blood loss≥200ml within 3 months, or plan to donate blood or blood components during the trial or within 1 month after the trial;
8. Individuals who have participated in any drug clinical trial within the past three months, including vaccine trials, and have received the investigational drug;
9. Individuals who have received any vaccination within the past month, or have a scheduled vaccination during the study, including inactivated vaccines, live attenuated vaccines, recombinant protein vaccines, recombinant adenovirus vaccines, RNA vaccines, DNA vaccines, etc.；
10. Individuals who have used any medication (including prescription drugs, over-the-counter medications, Chinese herbal medicines, or health supplements) within 14 days prior to the first dose，especially drugs or health products with anticoagulant, antiplatelet (such as aspirin) or fibrinolytic functions that increase the risk of bleeding;
11. Plan to undergo surgical procedures/operations or interventional procedures/operations with bleeding risks within 4 months after administration, including tooth extraction, thoracentesis, abdominal puncture, etc.;
12. Individuals with abnormal and clinically significant findings from physical examination, vital signs assessment, laboratory tests (including blood routine, urine routine, stool routine, occult blood, and blood biochemistry), chest radiographs, or abdominal ultrasound (hepatobiliary, pancreatic, spleen, and kidney assessments), which may affect the evaluation of study outcomes;
13. Individuals with abnormal heart rate, arrhythmias, or significant QTc interval prolongation (QTcF >450 ms for males and QTcF >470 ms for females) on 12-lead ECG, which could impact the interpretation of study results;
14. Individuals who test positive for infectious diseases, including hepatitis (B or C), HIV, and syphilis;
15. Individuals who test positive for alcohol on a breathalyzer;
16. Individuals who test positive for drug abuse (including morphine, methamphetamine, ketamine, tetrahydrocannabinolic acid, and methylenedioxymethamphetamine);
17. Individuals with a history of bleeding disorders or clear risk of coagulation dysfunction;
18. Male participants who refuse or are unable to use effective contraception from the time of informed consent until 3 months after the last follow-up. Effective contraceptive methods include vasectomy, consistent use of male condoms, partner's tubal ligation or hysterectomy, intrauterine device (IUD) insertion, etc.;
19. Individuals who, in the opinion of the investigator, have factors that would make them unsuitable for participation in the trial.

**Principle of Anti-KN060 Antibody Detection by Electrochemiluminescence (ECL):**

In this method, plasma samples are first treated with magnetic beads to remove endogenous FXI present in the samples. The magnetic bead–treated samples are then acidified to dissociate anti-KN060 antibodies from any KN060 drug that may be present in the sample. The dissociated antibodies are then captured on an ELISA-SA plate pre-coated with Bio-KN060. Next, another acidification step is performed to release the anti-KN060 antibodies bound to the ELISA-SA plate, and the released antibodies are transferred to an MSD plate for neutralization with an alkaline solution, allowing the anti-KN060 antibodies to adsorb onto the MSD plate. Finally, a Ru-KN060 mixture is added for incubation, forming a bridging complex of anti-KN060 antibody–Ru-KN060. After washing away unbound substances, an electric voltage is applied in a read buffer containing tripropylamine, generating an electrochemiluminescence signal. A signal is produced only when an anti-KN060 antibody–Ru-KN060 complex is formed.

**The abbreviations and definitions of the pharmacokinetic parameters are as follows:**

1. Area Under the Concentration-Time Curve from time zero to the last measurable concentration(AUC_0-t_): The area under the plasma concentration–time curve from dosing time to the time of the last measurable concentration; represents the extent of drug exposure actually measured.
2. Area Under the Concentration-Time Curve from time zero extrapolated to infinity(AUC_0-∞_): The area under the plasma concentration–time curve from dosing time extrapolated to infinity, including the extrapolated portion; used to estimate total drug exposure.
3. Mean Residence Time extrapolated to infinity(MRT_0-∞_): The mean time a drug molecule stays in the body, extrapolated to infinity.
4. Time to reach maximum plasma concentration(T_max_): The time to reach the maximum observed plasma concentration (Cmax); indicates the rate of absorption.
5. Maximum observed plasma concentration(C_max_): The maximum observed plasma concentration.
6. Elimination half-life(T_1/2_): The time required for the plasma drug concentration to decrease by half during the terminal elimination phase.
7. Clearance(CL): The volume of plasma from which the drug is completely removed per unit time.
8. Apparent Volume of Distribution(V_d_): A theoretical volume that relates the amount of drug in the body to the measured plasma concentration; indicates the extent of distribution.
9. Percentage of AUC extrapolated beyond the last measured point(AUC__% Extrap_): The percentage of the AUC₀₋∞ accounted for by the extrapolated portion beyond the last measured point.
10. Terminal Elimination Rate Constant(λz): The rate constant describing the terminal phase of drug elimination; used to calculate half-life and other parameters.

**TABLE S1**. Linear relationship analysis of dosage administration

| Parameters | Regression coefficients(β) | 90%CI |
| --- | --- | --- |
| 0.1 mg/kg~10.0 mg/kg | | |
| Ln(C_max_) | 0.987 | 0.94 - 1.04 |
| Ln(AUC_0-t_) | 1.124 | 1.07 - 1.17 |
| Ln(AUC_0-∞_) ^*^ | 1.046 | 0.99 - 1.10 |
| 1.0 mg/kg~10.0 mg/kg | | |
| Ln(C_max_) | 0.955 | 0.86 - 1.05 |
| Ln(AUC_0-t_) | 0.997 | 0.92 - 1.08 |
| Ln(AUC_0-∞_) | 0.987 | 0.91 - 1.07 |

Note: *The 0.1 mg/kg dose group was not included in the linear relationship analysis of AUC_0-∞_ due to abnormal PK parameters;90%CI，90%confidence interval.

**TABLE S2**. Correlation analysis between plasma concentration and pharmacodynamic biomarkers

| PD indicators | Correlation coefficien | | | | | | |
| --- | --- | --- | --- | --- | --- | --- | --- |
|  | 0.1 mg/kg | 0.3 mg/kg | 1.0 mg/kg | 2.5 mg/kg | 5.0 mg/kg | 10.0 mg/kg | Total of KN060 |
| APTT relative to baseline multiples | 0.935* | 0.745* | 0.667* | 0.520* | 0.531* | 0.405* | 0.623* |
| Percent change from baseline in FXI activity | - | - | -0.647* | -0.559* | -0.607* | -0.229* | -0.581* |
| Percent change from baseline in Free FXI | -0.963* | -0.714* | -0.767* | -0.588* | -0.518* | -0.309* | -0.499* |

Note: * indicates a P-value <0.05; Pearson correlation or Spearman rank correlation was used for correlation analysis; APTT relative to baseline multiples refers to the ratio of APTT values at each time point to the baseline APTT, expressed as a multiple; Percent change from baseline indicates the percentage difference of FXI activity and free FXI values from their baseline levels.

**TABLE S3.** Summary of Adverse Events by Grade and Type in Each Group

|  |  | 0.1 mg/kg  (N=2) | 0.3 mg/kg  (N=3) | 1.0 mg/kg  (N=6) | 2.5 mg/kg  (N=6) | 5.0 mg/kg  (N=6) | 10.0 mg/kg  (N=6) | Placebo (N=9) | Total (N=38) |
| --- | --- | --- | --- | --- | --- | --- | --- | --- | --- |
| Project |  | n (%) | n (%) | n (%) | n (%) | n (%) | n (%) | n (%) | n (%) |
| **AE** |  | 1 (50.0) | 3 (100.0) | 5 (83.3) | 6 (100.0) | 5 (83.3) | 6 (100.0) | 8 (88.9) | 34 (89.5) |
| **TEAE** |  | 1 (50.0) | 3 (100.0) | 5 (83.3) | 6 (100.0) | 5 (83.3) | 6 (100.0) | 8 (88.9) | 34 (89.5) |
| TEAEs of CTCAE Grade ≥2 |  | 0 | 0 | 2 (33.3) | 1 (16.7) | 1 (16.7) | 1 (16.7) | 1 (11.1) | 6 (15.8) |
| TEAEs of CTCAE Grade ≥3 |  | 0 | 0 | 1 (16.7) | 0 | 0 | 0 | 0 | 1 (2.6) |
| TEAEs Leading to Interruption/Adjustment of Medication |  | 0 | 0 | 0 | 0 | 0 | 0 | 0 | 0 |
| TEAEs Leading to Discontinuation of Medication |  | 0 | 0 | 0 | 0 | 0 | 0 | 0 | 0 |
| TEAEs Leading to Withdrawal from the Trial |  | 0 | 0 | 0 | 0 | 0 | 0 | 0 | 0 |
| TEAEs Leading to Death |  | 0 | 0 | 0 | 0 | 0 | 0 | 0 | 0 |
| **TRAE** |  | 1 (50.0) | 3 (100.0) | 5 (83.3) | 6 (100.0) | 5 (83.3) | 6 (100.0) | 8 (88.9) | 34 (89.5) |
| TRAEs of CTCAE Grade ≥2 |  | 0 | 0 | 2 (33.3) | 1 (16.7) | 1 (16.7) | 1 (16.7) | 1 (11.1) | 6 (15.8) |
| TrAEs of CTCAE Grade ≥3 |  | 0 | 0 | 0 | 0 | 0 | 0 | 0 | 0 |
| TRAEs Leading to Interruption/Adjustment of Medication |  | 0 | 0 | 0 | 0 | 0 | 0 | 0 | 0 |
| TRAEs Leading to Discontinuation of Medication |  | 0 | 0 | 0 | 0 | 0 | 0 | 0 | 0 |
| TRAEs Leading to Withdrawal from the Trial |  | 0 | 0 | 0 | 0 | 0 | 0 | 0 | 0 |
| TRAEs Leading to Death |  | 0 | 0 | 0 | 0 | 0 | 0 | 0 | 0 |
| **Adverse Events of Special Interest** |  | 0 | 0 | 0 | 0 | 0 | 0 | 0 | 0 |
| **SAEs** |  | 0 | 0 | 1 (16.7) | 0 | 0 | 0 | 0 | 1 (2.6) |
| **SAEs Related to the Trial Drug** |  | 0 | 0 | 0 | 0 | 0 | 0 | 0 | 0 |

Abbreviations: N = Number of subjects in each group; n = Number of subjects in each group meeting specific conditions; AE = Adverse Event; TEAE = Treatment-Emergent Adverse Event; TRAE = Treatment-Related Adverse Event; SAE = Serious Adverse Event.

Note: TEAEs are defined as any adverse events occurring after the administration of the trial drug. TRAEs are defined as TEAEs that are possibly unrelated, possibly related, probably related, or definitely related to the trial drug administration. Adverse events of special interest include bleeding and allergic reactions. SAEs are adverse medical events meeting one or more of the following severity criteria: resulting in death, life-threatening, requiring hospitalization or prolonging hospitalization, causing permanent or significant disability or organ function loss, causing congenital anomalies/birth defects, or other medically significant events. SAEs related to the trial drug are defined as SAEs that are possibly unrelated, possibly related, probably related, or definitely related to the study drug administration. Incidence is calculated using N as the denominator.

**TABLE S4.** PK, PD, and ADA Sample Collection Schedule*

| Number of Doses | Date | Dosing Time | | Time Window | PK Sample | PD Sample | | ADA Sample |
| --- | --- | --- | --- | --- | --- | --- | --- | --- |
|  |  |  |  |  |  | APTT/ FXI activity | Free FⅪ |  |
| 1 | D1 | Before Infusion Start | 30min~0min | - | 3 ml | 3ml | 3ml | 3 ml |
|  |  | After Infusion Start | 30min | ± 5 min | 3 ml | 3ml | 3ml |  |
|  |  | -- | Immediately After Infusion End | +5min | 3 ml | 3ml | 3ml |  |
|  |  | After Infusion Start | 2 h | ± 10 min | 3 ml | 3ml | 3ml |  |
|  |  |  | 4 h |  | 3 ml | 3ml | 3ml |  |
|  |  |  | 6h |  | 3 ml | 3ml | 3ml |  |
|  |  |  | 8 h |  | 3 ml | 3ml | 3ml |  |
|  |  |  | 12 h |  | 3 ml | 3ml | 3ml |  |
|  | D2 | After Infusion Start | 24h | ± 1 h | 3 ml | 3ml | 3ml |  |
|  | D3 |  | 48 h |  | 3 ml | 3ml | 3ml |  |
|  | D5 |  | 96 h |  | 3 ml | 3ml | 3ml |  |
|  | D7 |  | 144 h |  | 3 ml | 3ml | 3ml | 3 ml |
|  | D14 |  | 312 h | ± 1 d | 3 ml | 3ml | 3ml | 3 ml |
|  | D21 |  | 480h | ± 2 d | 3ml | 3ml | 3ml |  |
|  | D28 |  | 648 h | ± 2 d | 3 ml | 3ml | 3ml | 3 ml |
|  | D35 |  | 816h | ± 2 d | 3ml | 3ml | 3ml |  |
|  | D42 |  | 984 h | ± 3 d | 3 ml | 3ml | 3ml | 3 ml |
|  | D56 |  | 1320 h | ± 3 d | 3 ml | 3ml | 3ml | 3 ml |

*Starting from the second dose group, an additional sampling point at 6 hours post-infusion was included. Starting from the fifth dose group, sampling points on days 21 and 35 were added. Starting from the third dose group, additional time points for FXI activity sampling were included. FXI activity was not assessed in the first and second dose groups.


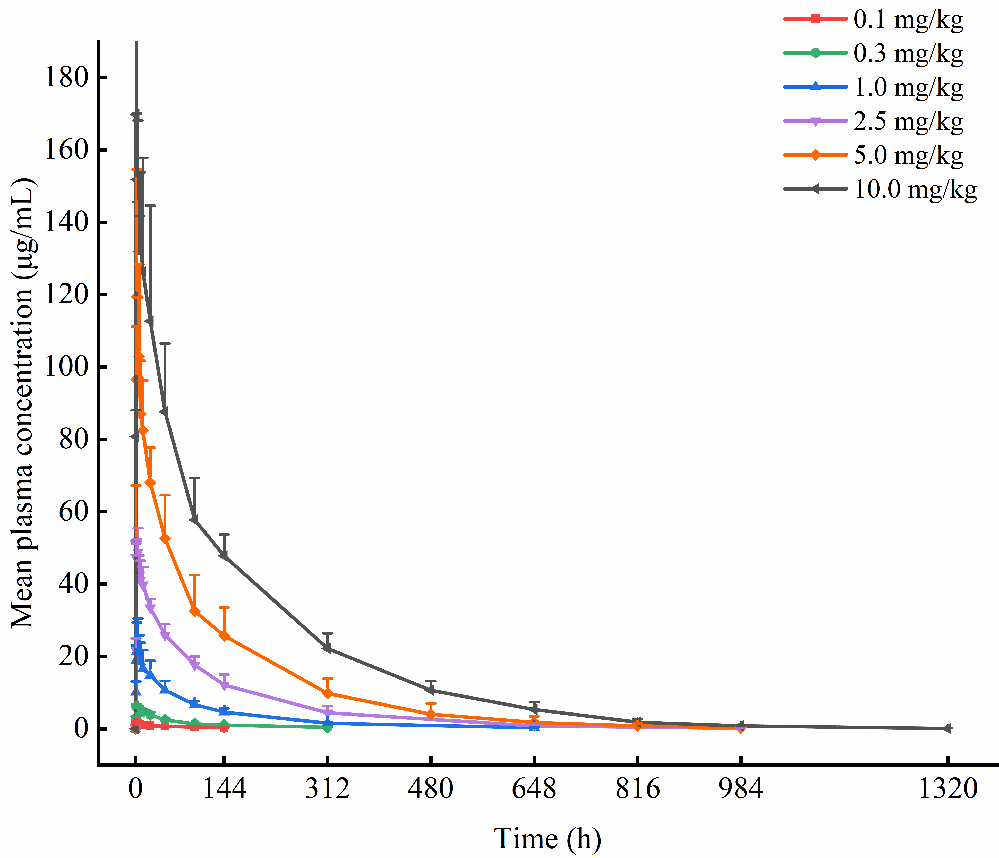


Fig.S1. Mean plasma concentration–time curves of KN060 for different dose groups ranging from 0.1 mg/kg to 10 mg/kg(linear scale). Healthy subjects received a single intravenous infusion of KN060 at doses from 0.1 mg/kg to 10.0 mg/kg. Blood samples were collected at the following time points: within 30 minutes before the infusion, 30 minutes after the start of the infusion, 5 minutes after the end of the infusion, and at 2, 4, 6, 8, 12, 24, 48, 96, and 144 hours after the start of the infusion, as well as on Days 14, 21, 28, 35, 42, and 56.


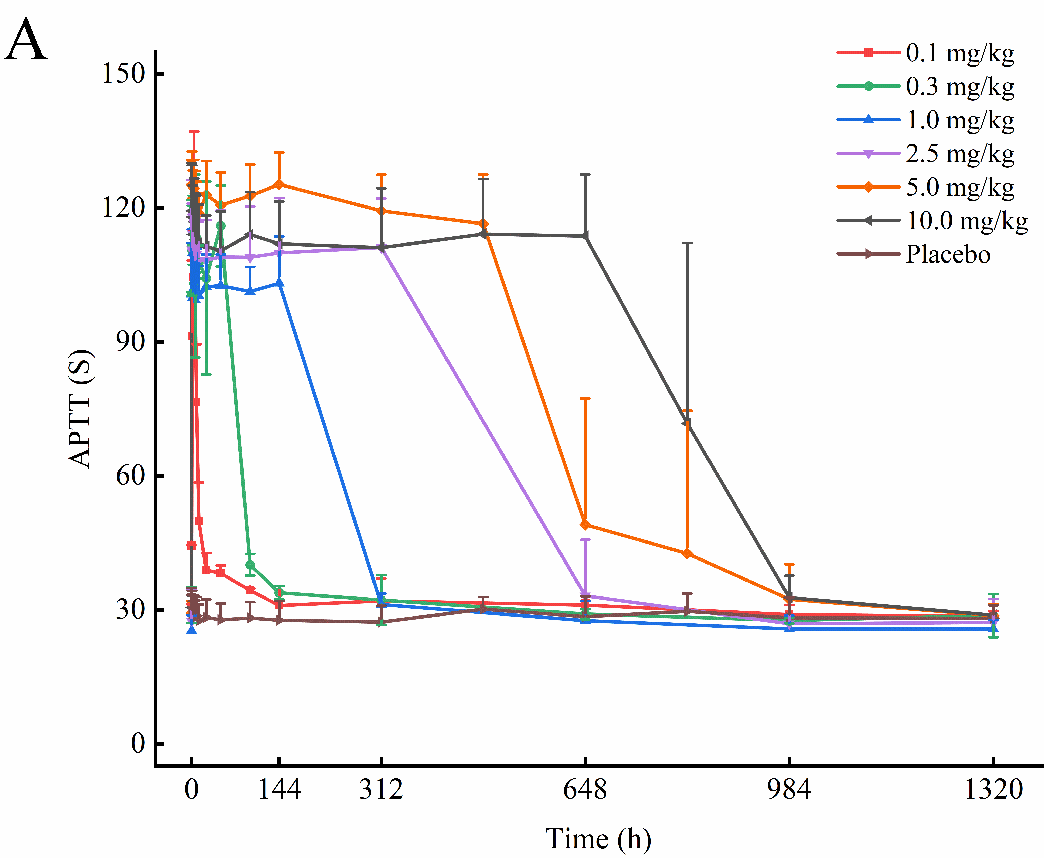


Fig.S2. Mean aPTT–time curves for KN060 and placebo in different dose groups (linear scale). Healthy subjects received a single intravenous infusion of KN060 at doses ranging from 0.1 mg/kg to 10.0 mg/kg.


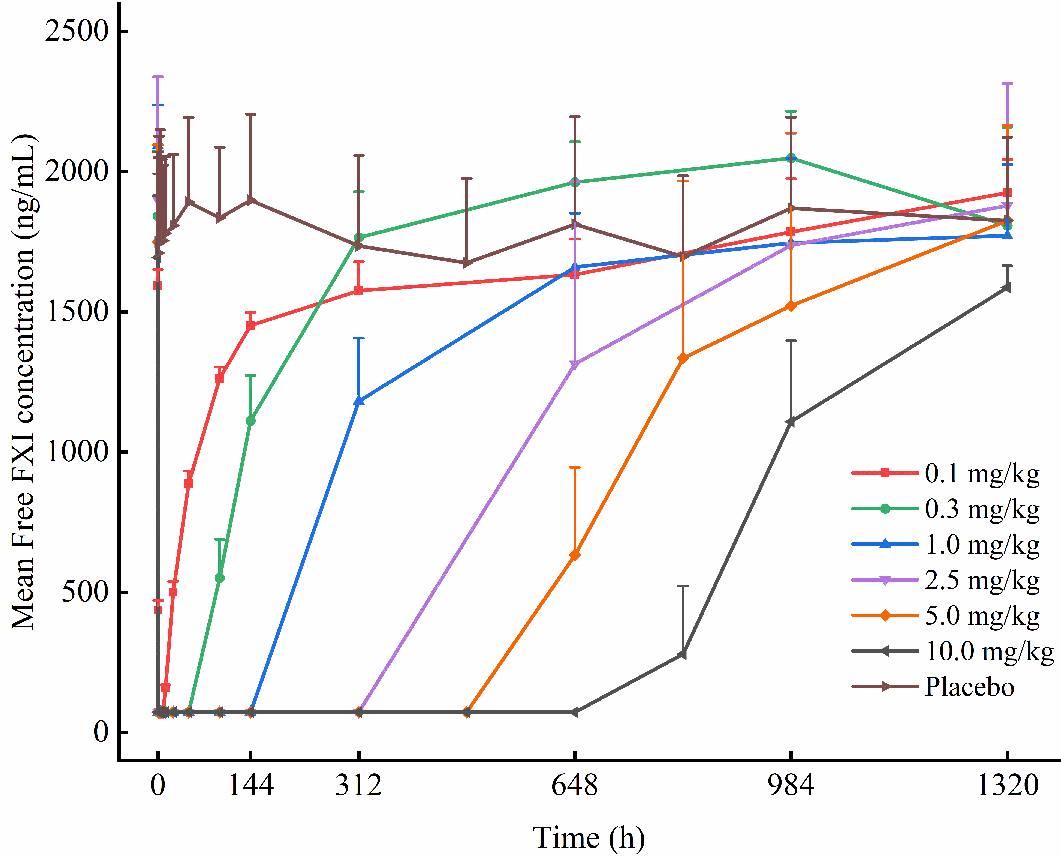


Fig.S3. Mean free FXI–time curves for KN060 and placebo in different dose groups (linear scale). Healthy subjects received a single intravenous infusion of KN060 at doses ranging from 0.1 mg/kg to 10.0 mg/kg. *BQL concentrations were assigned the LLOQ value (72.7 ng/mL).


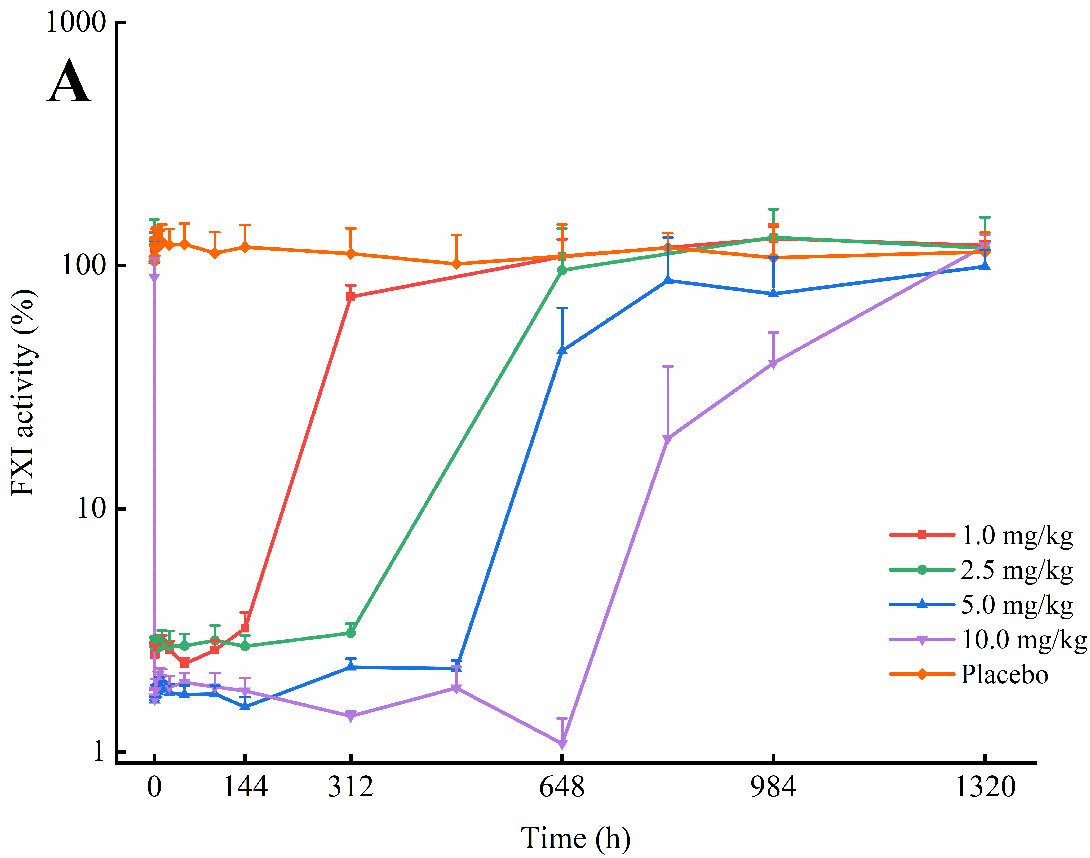


Fig.S4. Mean FXI activity–time curves for KN060 at 1, 2.5, 5, and 10 mg/kg and placebo (semi-logarithmic scale). FXI activity was suppressed to below 5% within 30 minutes after the start of the infusion.


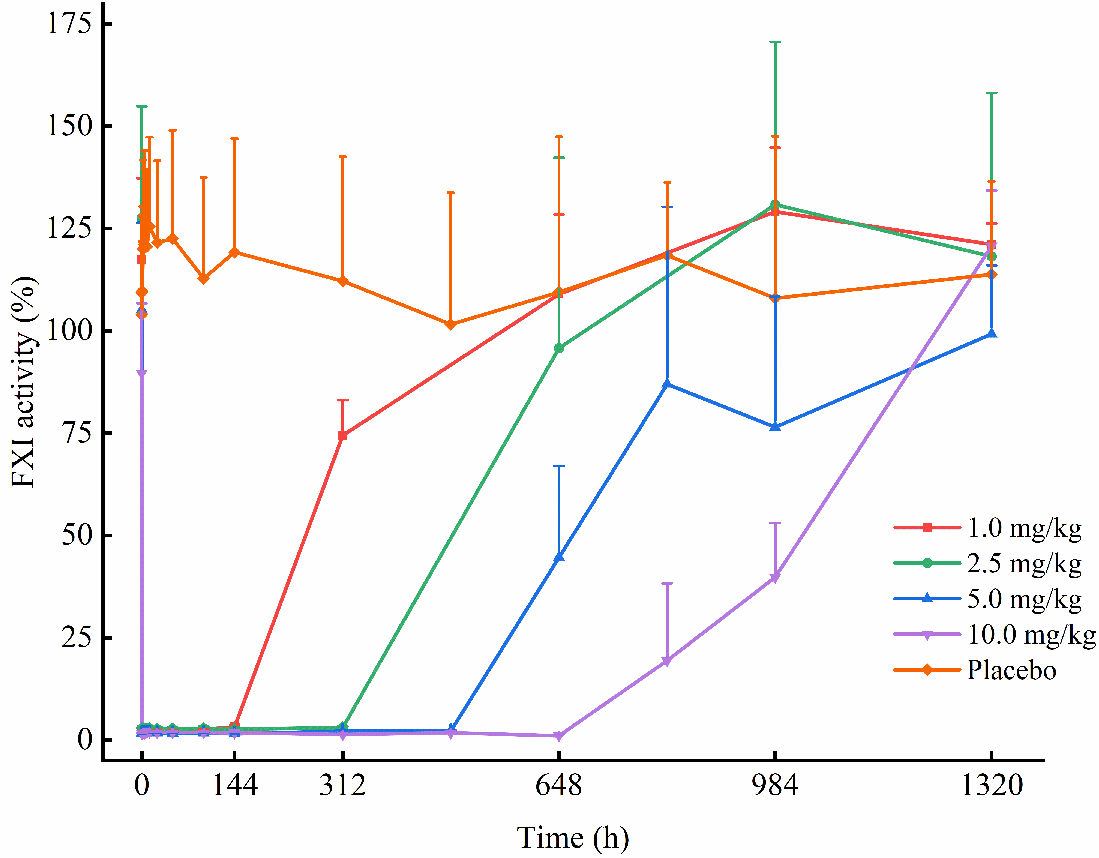


Fig.S5. Mean FXI activity–time curves for KN060 at 1, 2.5, 5, and 10 mg/kg and placebo (linear scale).FXI activity was suppressed to below 5% within 30 minutes after the start of the infusion.
