# Supplementary material for: Safety, tolerability, pharmacokinetics, and pharmacodynamics of KN060, a humanized anti-FXI/FXIa dual-domain antibody, following single ascending doses in healthy Chinese subjects
Source: Res Pract Thromb Haemost. 2025 Dec 29;10(1):103322. doi: 10.1016/j.rpth.2025.103322 (PMC12887396; doi:10.1016/j.rpth.2025.103322)
Supplement: CONSORT Diagram [file mmc2.docx]

**Figure 1: CONSORT 2025 Flow Diagram**

Flow diagram of the progress through the phases of a randomised trial of two groups (that is, enrolment, intervention allocation, follow-up, and data analysis)

Analysis

Follow-Up

Analysed for primary outcome (n=0 )

Excluded from analysis (give reasons) (n=8 )

Analysed for primary outcome (n=0 )

Excluded from analysis (give reasons) (n=8 )

Analysed for primary outcome (n=0 )

Excluded from analysis (give reasons) (n=8 )

Analysed for primary outcome (n=0 )

Excluded from analysis (give reasons) (n=8 )

Analysed for primary outcome (n=0 )

Excluded from analysis (give reasons) (n=8 )

Analysed for primary outcome (n=0 )

Excluded from analysis (give reasons) (n= 8)

Discontinued intervention (give reasons) (n=0 )

Lost to follow-up for primary outcome (give reasons) (n=0 ):

Discontinued intervention (give reasons) (n=0 )

Lost to follow-up for primary outcome (give reasons) (n=0 ):

Discontinued intervention (give reasons) (n=0 )

Lost to follow-up for primary outcome (give reasons) (n=0 ):

Discontinued intervention (give reasons) (n=0 )

Lost to follow-up for primary outcome (give reasons) (n=0 ):

Discontinued intervention (give reasons) (n=0 )

Lost to follow-up for primary outcome (give reasons) (n=0 ):

Discontinued intervention (give reasons) (n=0 )

Lost to follow-up for primary outcome (give reasons) (n=0 ):

Allocation

Allocated to intervention (n= 8)

Received allocated intervention (n=8 )

Did not receive allocated intervention (give reasons) (n=0 )

Allocated to intervention (n=8 )

Received allocated intervention (n=8 )

Did not receive allocated intervention (give reasons) (n=0 )

Allocated to intervention (n=8 )

Received allocated intervention (n= 8)

Did not receive allocated intervention (give reasons) (n=0 )

Allocated to intervention (n=8 )

Received allocated intervention (n=8 )

Did not receive allocated intervention (give reasons) (n=0 )

Allocated to intervention (n=4 )

Received allocated intervention (n=4 )

Did not receive allocated intervention (give reasons) (n=0 )

Allocated to intervention (n= 2)

Received allocated intervention (n=2 )

Did not receive allocated intervention (give reasons) (n=0 )

Excluded (n=325 )

Not meeting inclusion criteria (n=321 )

Declined to participate (n=4 )

Other reasons (n=0 )

Randomised (n=38 )

Enrolment

Assessed for eligibility (n=363 )
